# Supplementary material for: An Analysis of the Abstracts Presented at the Annual Meetings of the Society for Neuroscience from 2001 to 2006
Source: PLoS One. 2008 Apr 30;3(4):e2052. doi: 10.1371/journal.pone.0002052 (PMC2324197; doi:10.1371/journal.pone.0002052)
Supplement: Table S1 — Top 20 most frequently used words in each NCuts topic cluster. The words in each topic cluster are sorted in descending order of frequencies of usage, which are denoted in parenthesis. The size (or number of abstracts) of each cluster is also denoted in parenthesis. (0.05 MB DOC) [file pone.0002052.s001.doc]

| cluster1 (16729) | cluster2 (22647) | cluster3 (492) | cluster4 (7210) | cluster5 (19988) |
| --- | --- | --- | --- | --- |
| RAT (35194)  EFFECT (24743)  INCREAS (18525)  RECEPTOR (18236)  BEHAVIOR (17573)  ACTIV (17420)  STUDI (16337)  MICE (15485)  RESPONS (14499)  TEST (13670)  DAI (13660)  LEVEL (13420)  INDUC (13271)  EXPRESS (13088)  NEURON (12961)  RESULT (12808)  ANIM (11957)  SUGGEST (11898)  COCAIN (11049)  CONDITION (10875)  STRESS (10613)  MALE (10479)  BRAIN (10474)  CONTROL (10323)  GROUP (10319)  SHOW (9717)  INJECT (9192)  DOSE (8847)  TREATMENT (8727)  DRUG (8530) | NEURON (46263)  CELL (42688)  RECEPTOR (37180)  ACTIV (34164)  EXPRESS (22323)  SYNAPT (19458)  INCREAS (18339)  PROTEIN (17543)  CURRENT (17382)  CHANNEL (17248)  STUDI (16065)  EFFECT (15910)  RESULT (15274)  SUGGEST (15125)  RESPONS (13726)  POTENTI (13648)  RAT (13188)  FUNCTION (13034)  INDUC (13027)  DEPEND (11601)  STIMUL (11275)  SHOW (11113)  RECORD (10984)  DENDRIT (10845)  SUBUNIT (10774)  TYPE (10385)  SYNAPS (10108)  INHIBIT (9858)  ROLE (9712)  REGUL (9394) | SONG (2343)  NEURON (941)  HVC (910)  BIRD (804)  MALE (701)  EXPRESS (640)  ACTIV (629)  VOCAL (614)  AUDITORI (611)  RESPONS (581)  FINCH (551)  BRAIN (520)  ZEBRA (499)  LEARN (474)  FEMAL (462)  AREA (450)  SING (426)  ADULT (417)  RA (395)  SUGGEST (368)  CONTROL (367)  NUCLEU (367)  CELL (344)  RESULT (341)  SYSTEM (336)  SHOW (330)  PATTERN (326)  BEHAVIOR (322)  SONGBIRD (312)  RECORD (300) | NEURON (10964)  RAT (10326)  ACTIV (9731)  SPINAL (9305)  PAIN (8039)  RECEPTOR (7664)  NERV (7396)  EFFECT (7110)  RESPONS (6779)  INCREAS (6740)  STUDI (6577)  MUSCL (6160)  CORD (6001)  INDUC (5712)  CELL (5568)  EXPRESS (5317)  RESULT (5293)  INJECT (4855)  STIMUL (4781)  SUGGEST (4528)  MECHAN (4338)  CONTROL (4188)  INJURI (4033)  DORSAL (3996)  DAI (3852)  MORPHIN (3795)  MICE (3556)  SHOW (3377)  LEVEL (3311)  ANIM (3290) | CELL (60997)  NEURON (40579)  EXPRESS (32151)  ACTIV (22822)  BRAIN (18671)  INCREAS (18271)  PROTEIN (18127)  INDUC (17830)  STUDI (16532)  GENE (16290)  RAT (15737)  MICE (15322)  EFFECT (14642)  RESULT (14385)  SUGGEST (12617)  LEVEL (11862)  SHOW (10982)  CULTUR (10566)  FUNCTION (10301)  RECEPTOR (10113)  DEATH (9681)  DEVELOP (9596)  DAI (9585)  ROLE (9444)  INJURI (9412)  CONTROL (9396)  REGUL (9337)  FACTOR (9058)  TREATMENT (9032)  DISEAS (9025) |
| cluster6 (3609) | cluster7 (736) | cluster8 (794) | cluster9 (14192) | cluster10 (1146) |
| AD (6270)  MICE (5479)  PROTEIN (4835)  AMYLOID (4800)  CELL (4787)  BRAIN (4585)  APP (4552)  DISEAS (4257)  TAU (4164)  NEURON (4063)  ACTIV (3911)  EXPRESS (3503)  ALZHEIM (3247)  STUDI (3205)  INCREAS (3205)  LEVEL (3200)  AG (2601)  RESULT (2526)  SUGGEST (2487)  PEPTID (2389)  EFFECT (2313)  PLAQU (2277)  SECRETAS (2155)  TRANSGEN (2155)  SHOW (2126)  HUMAN (1981)  ABETA (1885)  BETA (1834)  CONTROL (1773)  DEPOSIT (1740) | STUDENT (1796)  NEUROSCI (1197)  BRAIN (1126)  DATA (822)  RESEARCH (811)  DEVELOP (617)  PRESENT (532)  LEARN (528)  PROGRAM (463)  STUDI (457)  SCIENC (439)  INCLUD (429)  MODEL (427)  EXPERI (427)  SCHOOL (419)  SYSTEM (417)  BEHAVIOR (388)  ACTIV (387)  BASE (382)  PROVID (381)  INFORM (359)  EDUC (345)  IMAG (334)  FUNCTION (333)  UNDERGRADU (332)  NEURON (327)  DESIGN (297)  YEAR (297)  TEACH (288)  UNIVERS (281) | CIRCADIAN (2044)  SCN (1724)  LIGHT (1648)  ACTIV (1257)  RHYTHM (1247)  EXPRESS (1236)  PHASE (1057)  CELL (1036)  CLOCK (1024)  NEURON (877)  DAI (848)  RAT (748)  MICE (678)  DARK (647)  GENE (642)  EFFECT (639)  TIME (630)  STUDI (599)  RESPONS (594)  CYCL (587)  RESULT (585)  RECEPTOR (560)  ANIM (552)  NUCLEU (521)  REGUL (515)  SHOW (511)  INDUC (509)  LEVEL (491)  NIGHT (489)  SUGGEST (466) | ACTIV (22742)  RESPONS (17698)  TASK (16343)  NEURON (15088)  VISUAL (14281)  SUBJECT (14164)  STUDI (12833)  RESULT (11355)  CORTEX (10766)  MOVEM (10446)  PERFORM (10161)  AREA (10012)  SHOW (9480)  PRESENT (9169)  CONTROL (9069)  STIMULI (9032)  TIME (8880)  TARGET (8642)  SUGGEST (8486)  PROCESS (8468)  FUNCTION (8393)  STIMULU (8284)  EFFECT (7860)  REGION (7793)  INCREAS (7697)  TRIAL (7561)  CONDITION (7405)  BRAIN (7208)  CELL (7192)  RECORD (7112) | SLEEP (5108)  ACTIV (2364)  NEURON (1985)  RAT (1710)  INCREAS (1585)  WAKE (1567)  REM (1296)  RECORD (1105)  EFFECT (948)  STUDI (928)  STATE (924)  RESULT (824)  EEG (806)  CELL (801)  MICE (739)  SUGGEST (721)  CONTROL (712)  DAI (710)  SHOW (690)  DECREAS (652)  LEVEL (642)  RECEPTOR (642)  DEPRIV (635)  RESPONS (625)  BRAIN (612)  BEHAVIOR (598)  PERIOD (597)  ANIM (594)  WAVE (583)  INDUC (582) |
